# Tris(2-chloroethyl) Phosphate (TCEP) Elicits Hepatotoxicity by Activating Human Cancer Pathway Genes in HepG2 Cells

**DOI:** 10.3390/toxics8040109

**Published:** 2020-11-20

**Authors:** Abdullah M. Al-Salem, Quaiser Saquib, Maqsood A. Siddiqui, Javed Ahmad, Abdulaziz A. Al-Khedhairy

**Affiliations:** 1 Zoology Department, College of Sciences, King Saud University, P.O. Box 2455, Riyadh 11451, Saudi Arabia; alsalem1985@hotmail.com (A.M.A.-S.); kedhairy@ksu.edu.sa (A.A.A.-K.); 2 DNA Research Chair, Zoology Department, College of Sciences, King Saud University, P.O. Box 2455, Riyadh 11451, Saudi Arabia; masiddiqui@ksu.edu.sa (M.A.S.); javedbiochem@gmail.com (J.A.)

**Keywords:** tris(2-chloroethyl) phosphate, TCEP, transcriptome, organophosphorus flame retardants, apoptosis, HepG2

## Abstract

Tris(2-chloroethyl) phosphate (TCEP) is one of the organophosphorus flame retardants (OPFRs) used in consumer commodities and have been detected in human body fluids. Research on TCEP-induced transcriptomic alterations and toxicological consequences in liver cells is still lacking. Herein, human hepatocellular (HepG2) cells were treated with 100, 200, and 400 μM TCEP for 3 days to quantify hepatotoxicity by MTT, NRU, and comet assays. Apoptosis, mitochondrial membrane potential (*ΔΨm*), oxidative stress, and Ca^2+^ influx were measured by flow cytometry. A qPCR array was employed for transcriptomic analysis. MTT and NRU data showed 70.92% and 75.57% reduction in cell survival at 400 μM. In addition, 20-fold greater DNA damage was recorded at 400 μM. Cell cycle data showed 65.96% subG1 apoptotic peak in 400 μM treated cells. An elevated level of oxidative stress, esterase, Ca^2+^ influx, and *ΔΨm* dysfunction were recorded in TCEP-treated cells. Out of 84 genes, the qPCR array showed upregulation of 17 genes and downregulation of 10 key genes belonging to human cancer pathways. Our study endorses the fact that TCEP possesses hepatotoxic potential at higher concentrations and prolonged exposure. Hence, TCEP may act as a cancer-inducing entity by provoking the gene network of human cancer pathways.

## 1. Introduction

Tris(2-chloroethyl) phosphate (TCEP) is one of the organophosphorus flame retardants (OPFRs) widely used as plasticizer, glue, or lacquer for the manufacturing of varnish, plastics, floor polish, foams, and furniture [1]. Widespread application of TCEP in consumer commodities has verified its abundance in the environment, residential areas, as well as indoor household settings. TCEP has been predominantly reported within the indoor environment, such as houses, workplaces, and student dormitories, in Germany and China [2,3]. A new source of TCEP exposure has been affirmed by the presence of flame retardants in smartphones, revealing that phone cases have significant (5.2 × 10^2^ ng/g) amounts of TCEP [4]. Additionally, dust samples collected from car cabins in Japan showed 71 μg g^−1^ of TCEP. Hence, humans are at risk of TCEP exposure by inhalation and ingestion of dust from contaminated car cabins or the air [5].

A case–control study on humans exposed to household dusts containing TCEP showed an increased risk for the development of clinically significant papillary thyroid cancer [6]. During 2011–2015, the unregulated use of TCEP has led to its detection in the human body, ranging from 536 to 605 ng/g lipid weight [7]. More importantly, TCEP has been detected in the breast milk of Australian lactating mothers (<0.13 to 0.47 ng/mL). Assuming a daily intake of 450 mL of breast milk by infants less than 1 year old, the average estimated intake of TCEP is determined to be 4.6 ng/kg/d [8]. Along the same line, TCEP and its metabolites have been detected in pregnant women in the USA [9]. A biomonitoring study on 323 individuals in China from different age groups showed the presence of TCEP and its metabolites in urine, leading to calculated exposure limits of TCEP from 96.9 to 46,700 (equivalent to 52.2–25,200) ng/kg bw/d [10]. Within the healthcare environment, intensive care unit patients subjected to treatment with specific medical devices have also exhibited urinary presence of TCEP [11]. Not only are humans at risks of TCEP exposure; rather, a recent environmental study also categorized TCEP as the most frequently detected OPFR in the bays of China and Japan [12]. Water samples of different grades (raw, finished, and tap water) collected from drinking water plants in China also confirmed the presence of TCEP. Consequently, TCEP has been recognized as “to be carcinogenic” and labeled as carcinogen category 2, highly toxic, and persistent in the environment.

Concerning animal toxicity, in vivo studies on TCEP exhibited cancer induction along with testicular damage, which led to impairment of the reproductive system in rats [13,14]. Furthermore, SD rats exposed to TCEP (50–250 mg/kg bw) for 60 days triggered metabolomic changes along with neurological alterations viz. decreased spatial learning, memory function, and increased apoptosis and necrotic lesions in the brain cells. Invasion of inflammatory cells and calcified/ossified foci formation were also observed in the cortex region of exposed rats [15].

In vitro studies on the toxic effects of TCEP in liver cell lines are still elusive. We have encountered very few reports on the hepatotoxicity of TCEP. For example, human hepatoma (Hep3B) cells exposed to TCEP under in vitro conditions demonstrated reduced cell survival and senescence like growth arrest via the p21^Waf1/Cip1^-Rb pathway [16]. Along the same line, the TCEP-exposed rat hepatic (H4IIE) cell line showed decreased cell survival at higher concentrations (50–100 μM). Moreover, H4IIE enzymatic activities (EROD, MROD, CAT, and GPX) were augmented after TCEP exposure [17]. Human hepatocellular (HepG2) cells exposed to a combined mixture of TCEP and benzo (a) pyrene demonstrated the release of inflammatory responses (IL-6 and IL-8) via activation of the EGFR-ERK1/2 pathway [18]. RNA sequencing (RNA-Seq) of HepG2 cells exposed to TCEP at sublethal concentrations affected several genes related to xenobiotic metabolism, immune responses, and steroid hormone biosynthesis [19]. Furthermore, HepG2 cells exposed to TCEP demonstrated cytotoxicity and growth arrest through attenuation of the SIRT1-independent PI3K/Akt/mTOR pathway [20]. On the other hand, in vitro studies on non-hepatic cells also confirmed the toxicity of TCEP in human lymphocytes [21]. Human alveolar basal epithelial (A549) cells exposed to TCEP (50–300 μM) for 48 h revealed a sizeable amount of cellular toxicity, as well as mitochondrial damage, leading to apoptosis via the caspase pathway [22]. In addition, O-linked N-acetylglucosamine (O-GlcNAc) transferase activity in neuronal cells (PC12) profoundly declined to 30% upon exposure to 100 μM of TCEP [23].

Despite the inhalation route, dust containing TCEP could also be ingested and metabolized to cause organ toxicity, including hepatic anomalies [5,13,14]. However, to date, no study has been conducted to delineate the cross-talk between the array of metabolic and signaling genes playing crucial roles in TCEP-induced hepatotoxicity and induction of cancer pathways in human hepatic cells. Such a viewpoint on TCEP has been assessed in the current study, providing novel data to bridge this information gap. We used HepG2 cells due to their high differentiating ability and genotypic resemblance to normal liver cells. Such characteristics of HepG2 make it a preferred cell line for the toxicological screening of chemicals [24]. Toxicological consequences in HepG2 cells were measured by exposing them to 100 to 400 μM of TCEP for 3 days. Cytotoxicity was quantified by MTT and NRU assays. DNA strand breaks were quantified by the comet assay. Flow cytometric analyses were done for cell death, oxidative stress, and mitochondrial membrane potential (*ΔΨm*). Transcriptomic alterations were analyzed by qPCR array plates containing 84 genes belonging to the network of human cancer pathways.

## 2. Materials and Methods

### 2.1. Cell Culture and Selection of TCEP Exposure Concentrations

MTT and NRU assays were performed to quantify the cytotoxicity of tris(2-chloroethyl) phosphate (TCEP) (purity 97%, Cat. No. 119660, CAS No. 115-96-8, Sigma-Aldrich, St. Louis, MO, USA) in HepG2 cells. The HepG2 cell line was obtained from American Type Culture Collection (ATCC) (Beltsville, MD, USA). Selection of TCEP exposure concentrations and duration of exposure were first analyzed in pilot experiments by treating the HepG2 cells for 1 to 3 days in an incomplete RPMI-1640 medium supplemented with low (5, 10, 25, 50 μM) and high (100, 200, 400 μM) concentrations of TCEP in a CO_2_ incubator (5%, 95% humidity) at 37 °C (Hera Cell 150i, Thermo Scientific, Langenselbold, Germany). No cytotoxic effects were found at the above-mentioned concentrations after 2 days of exposure (data not shown). Only the higher concentrations (100, 200, 400 μM) of TCEP showed significant cytotoxicity after 3 days. Consequently, these concentrations were selected for further experiments.

### 2.2. MTT and NRU Cytotoxicity Assays

Cytotoxic effects of TCEP in HepG2 cells were quantitated by following our previously described method [25]. For the MTT assay, HepG2 cells were seeded in 96-well plates and allowed to grow for overnight at 37 °C in a CO_2_ incubator (5%) having 95% humidity. On the next day, cell culture medium was disposed under sterile conditions. Fresh incomplete RPMI-1640 media containing varying concentrations (100, 200, and 400 μM) of TCEP were dispensed into the pre-designated wells. Untreated control wells were replenished with fresh incomplete RPMI-1640 medium only. Subsequently, cells were allowed to grow for 3 days in a CO_2_ incubator (5%, 95% humidity) at 37 °C. After completion of 3 days, the culture medium was discarded, and cells were washed with Ca^2+^- and Mg^2+^-free phosphate-buffered saline (PBS). Thereafter, 5 mg/mL of MTT dye prepared in cell culture medium was dispensed in all wells, and plates were incubated for 4 h at 37 °C. After the specified time of incubation, the dye-containing culture medium was aspirated from each well and discarded. DMSO (200 µL) was then dispensed in all wells to dissolve the crystals of insoluble formazan, whose absorbance was measured at 550 nm on a microplate reader (Multiskan Ex, Thermo Scientific, Shanghai, China).

TCEP-induced lysosomal fragility, as an indicator of cytotoxicity, was quantitated by NRU assay following a previously described method [25]. For the NRU assay, HepG2 cells were seeded in 96-well plates and allowed to grow overnight at 37 °C in a CO_2_ incubator (5%, 95% humidity). Subsequently, old cell culture media from the wells of overnight-grown HepG2 cells were discarded. Fresh medium (incomplete RPMI-1640) containing TCEP (100, 200, and 400 μM) was dispensed into their pre-designated wells. Under similar conditions, only incomplete RPMI-1640 medium was added to untreated control wells. HepG2 cells were allowed to grow for 3 days at 37 °C in a CO_2_ incubator (5%, 95% humidity). After 3 days, cell culture medium was discarded, and cells were washed with Ca^2+^- and Mg^2+^-free PBS. Neutral red (NR) dye (50 μg/mL) freshly prepared in an incomplete RPMI-1640 medium was added in all wells, and the cells were incubated with NR dye for 3 h at 37 °C in a CO_2_ incubator (5%, humidity 95%). Later, NR dye was discarded, and cells were washed with solution containing calcium chloride (1%) and formaldehyde (0.5%). HepG2 cells were additionally incubated for 20 min at 37 °C in 1% acetic acid and 50% ethanol solution to extract the dye. Finally, absorbance of red color developed in the wells was measured at 550 nm on a microplate reader [25].

### 2.3. Alkaline Comet Assay

TCEP-induced DNA damage in HepG2 cells was quantitated by following the previously described method for alkaline comet assay [26]. Firstly, HepG2 cells were seeded in 24-well plates and grown at 37 °C overnight in a CO_2_ incubator (5%, 95% humidity). The next day, cell culture medium was disposed from the wells, and fresh incomplete RPMI-1640 media containing 100, 200, and 400 μM of TCEP were dispensed into their pre-designated wells. Untreated control wells were replenished with incomplete RPMI-1640 medium only. HepG2 cells were then allowed to grow in the presence of TCEP for 3 days at 37 °C in a CO_2_ incubator (5%, 95% humidity). For the positive control, HepG2 cells were exposed to incomplete RPMI-1640 medium containing ethyl methanesulfonate (EMS) (2 mM) for 2 h in a CO_2_ incubator (5%, 95% humidity), and then they were processed for comet slide preparation in a similar way as described in subsequent steps. After 3 days of exposure, HepG2 cells were detached using trypsin EDTA (0.1%), and cell pellets were obtained by spinning the cells at 1000 rpm for 3 min. Old cell culture media from the wells were aspirated and thrown away. Cells were washed twice by Ca^2+^- and Mg^2+^-free PBS (1000 rpm, 3 min). Subsequently, 100 µL of PBS was added in each tube to suspend the cells, and 100 µL of 1% low meting agarose (LMA) was added to tubes one after another. For making a single comet slide, 80 µL of cell-agarose mixture from the tube was aspirated and overlaid on one-third frosted end slide, precoated with 1% normal meting agarose (NMA), followed by rapidly placing a glass cover slip (24 × 60 mm, No. 1). Duplicate slides from each concentration and controls were prepared by the above-described process, and all slides were kept on ice to allow proper gelling of agarose. Thereupon, coverslips were gently removed, and 90 µL of LMA (0.5%) was overlaid on each slide and left on ice for gel solidification. Cell membranes from the imbedded cells were degenerated by immersing the slides in a lysis buffer (NaCl 2.5 M, EDTA 100 mM, Tris 10 mM, Triton X100 1%; pH 10) for 24 h at 4 °C. All slides were submerged in an electrophoresis buffer (300 mM NaOH, 1 mM EDTA; pH > 13) for 20 min, and electrophoresis was done at 24 V for 30 min. Post electrophoresis, neutralization buffer (Tris-HCl 0.4 mM; pH 7.5) was added on the slides for 5 min (3 repeats), and DNA was stained using ethidium bromide solution (20 μg/mL) [26]. Quantification of DNA damage was done randomly in 50 cells/slide/concentration, and comet imaging was carried out using a fluorescence microscope (Eclipse 80i, Tokyo, Japan) with Comet Assay IV software (Perceptive Instruments, Bury St Edmunds, UK).

### 2.4. Flow Cytometric Studies

#### 2.4.1. Cell Cycle Analysis

Cell cycle dysfunction was analyzed by following the previously described method [27]. In brief, HepG2 cells were seeded in 24-well plates overnight at 37 °C in a CO_2_ incubator (5%, 95% humidity). On the next day, cell culture medium from the wells was discarded, followed by replenishment with incomplete RPMI-1640 medium containing 100, 200, and 400 μM of TCEP. Control cells received fresh incomplete RPMI-1640 medium only. Cells were then allowed to grow in the presence of TCEP for 3 days at 37 °C in a CO_2_ incubator (5%, 95% humidity). After the specified days of incubation, cells were detached, and pellets were obtained by centrifugation at 1000 rpm for 3 min. Culture medium was discarded, and washing was done twice with cold PBS. Cells pellets were suspended in 500 μL of chilled ethanol solution (70%) and kept on ice for 1 h. Afterwards, cells were centrifuged at 1000 rpm for 3 min, and ethanol was discarded. Pellets were finally suspended in 500 μL of staining dye (RNase A 0.05 mg/mL; propidium iodide 50 µg/mL; Triton X100 0.1%). Cells were stained for 1 h at room temperature. A total of 10,000 cells were read at FL-3 channel via 675 band-pass filter on a flow cytometer (Coulter Epics XL/Xl-MCL, Miami, USA). Cell cycle changes were analyzed by Coulter Epics XL/XL-MCL, System II Software, Version 3.0 [27].

#### 2.4.2. Ca^2+^ Influx and Mitochondrial Membrane Potential (*ΔΨm*)

Ca^2+^ influx and mitochondrial membrane potential (*ΔΨm*) in TCEP-exposed cells were measured following the previously described method [28]. Before commencing the experiments, HepG2 cells were seeded separately in 24-well plates and allowed to grow overnight at 37 °C in a CO_2_ incubator (5%, 95% humidity). On the next day, cell culture medium was aspirated and discarded under sterile conditions. Fresh incomplete RPMI-1640 media containing 100, 200, and 400 μM of TCEP were dispensed in their pre-designated wells. Control wells received only incomplete RPMI-1640 medium. Subsequently, HepG2 cells were allowed to grow for 3 days in the presence of TCEP at 37 °C in a CO_2_ incubator (5%, 95% humidity). After 3 days of incubation, cells were detached, and cell pellets were obtained by centrifugation at 1000 rpm for 3 min. Pellets were subjected to washing by PBS, and finally cells were suspended in PBS (500 μL). For the quantification of Ca^2+^ influx, HepG2 cells were incubated with 4 µM of Fluo-3 dye for 1 h at 37 °C in a CO_2_ incubator (5%, 95% humidity). For quantifying changes in *ΔΨm*, cells were stained with 5 µg/mL of Rh123 dye for 1 h at 37 °C in a CO_2_ incubator (5%, 95% humidity). Post staining, the fluorescence of 10,000 cells was recorded at 530 nm on a log scale (FL-1) with a flow cytometer [28].

#### 2.4.3. Reactive Oxygen Species (ROS), Nitric Oxide (NO), and Esterase Activities

Intracellular ROS, NO, and esterase level quantifications were done following the previously described method [28]. Firstly, HepG2 cells were seeded separately in 24-well plates and allowed to grow overnight in a CO_2_ incubator (5%, 95% humidity) maintained at 37 °C. Later, cell culture medium was aspirated and discarded under sterile conditions. Fresh incomplete RPMI-1640 media containing 100, 200, and 400 μM of TCEP were dispensed in their pre-designated wells to expose the HepG2 cells for 3 days at 37 °C in a CO_2_ incubator (5%, 95% humidity). After the specified time of incubation, cell culture medium was discarded, and cells were washed using PBS. Cell pellets were then suspended in 500 µL of PBS and processed separately for ROS, NO, and esterase level quantifications on a flow cytometer. For ROS analysis, 5 µM DCFH-DA dye was added to cell suspensions. NO was quantitated by the addition of DAF2-DA (5 µM) dye. Esterase quantification was done by staining the cells with 5 µM of CFDA-SE dye. HepG2 cells were allowed to separately stain with the above dyes for 1 h (37 °C) in a CO_2_ incubator (5% CO_2_). After the specified incubation time, 10,000 cells were analyzed on a flow cytometer at FL-1 channel (530 nm) [28].

### 2.5. Transcriptomic Analysis by qPCR Array

qPCR array experiments were done to quantify the transcriptomic alterations by following the previously described methods [27,28]. For the qPCR array, HepG2 cells were first seeded in 6-well plates and allowed to grow overnight in a CO_2_ incubator (5%, 95% humidity) maintained at 37 °C. Subsequently, cell culture medium was disposed, and incomplete RPMI-1640 medium containing 100 μM of TCEP was replenished in the replicate wells. Control wells were dispensed with incomplete RPMI-1640 medium only. HepG2 cells were allowed to grow for 3 days in a CO_2_ (5%) incubator with 95% humidity. After the specified exposure days, the culture plate was taken out, and cell culture medium was discarded. Following the manufacturer’s protocol, total RNA was isolated from control and TCEP-treated groups using RNeasy Mini Kit (Cat. No. 74106, Qiagen, Hilden, Germany). RNA purity was determined on a Nanodrop spectrophotometer (Nanodrop 8000, Thermo Scientific, Waltham, MA, USA). Total RNA (1 μg) was used for cDNA synthesis using the RT^2^ first strand synthesis kit (Cat. No. 330404, Hilden, Germany) following the manufacturer’s recommended protocol. The array plates containing 84 genes linked to human cancer pathways were employed to quantify the transcriptomic changes in TCEP-exposed HepG2 cells. For the RT^2^ Profiler™ PCR Array experiments (96-well format containing 84 genes, Cat # PAHS-033Z, Qiagen, MD, USA), the reaction mixture (20 μL) containing 1 μg of cDNA was dispensed in each well. Transcriptional variations were recorded on Roche^®^ LightCycler^®^ 480 real-time PCR (Roche Diagnostics, Basel, Switzerland) following the manufacturer’s cycling programs. *ACTB*, *GAPDH*, *RPLP0*, *HPRT1*, and *B2M* were the five house-keeping genes used for the normalization and data analysis between TCEP-treated HepG2 cells and control (^Δ^Ct) HepG2 cells using the online platform (Gene Globe) provided by Qiagen [27,28]. Genes exhibiting fold-changes >2.0 (*p* < 0.05) (upregulation or downregulation) were considered significant.

### 2.6. Statistical Analysis

Statistical significance was determined first via data normality (Kolmogorov and Smirnov’s test) and homogeneity (Bartlett’s test) of variance. Data were then examined by one-way ANOVA using Dunnett’s multiple comparisons method using Sigma Plot 14.0, USA. *p* < 0.05 was the level of statistical significance, unless otherwise stated.

## 3. Results

### 3.1. TCEP Induced Cytotoxicity in HepG2 Cells

**Figure 1:**
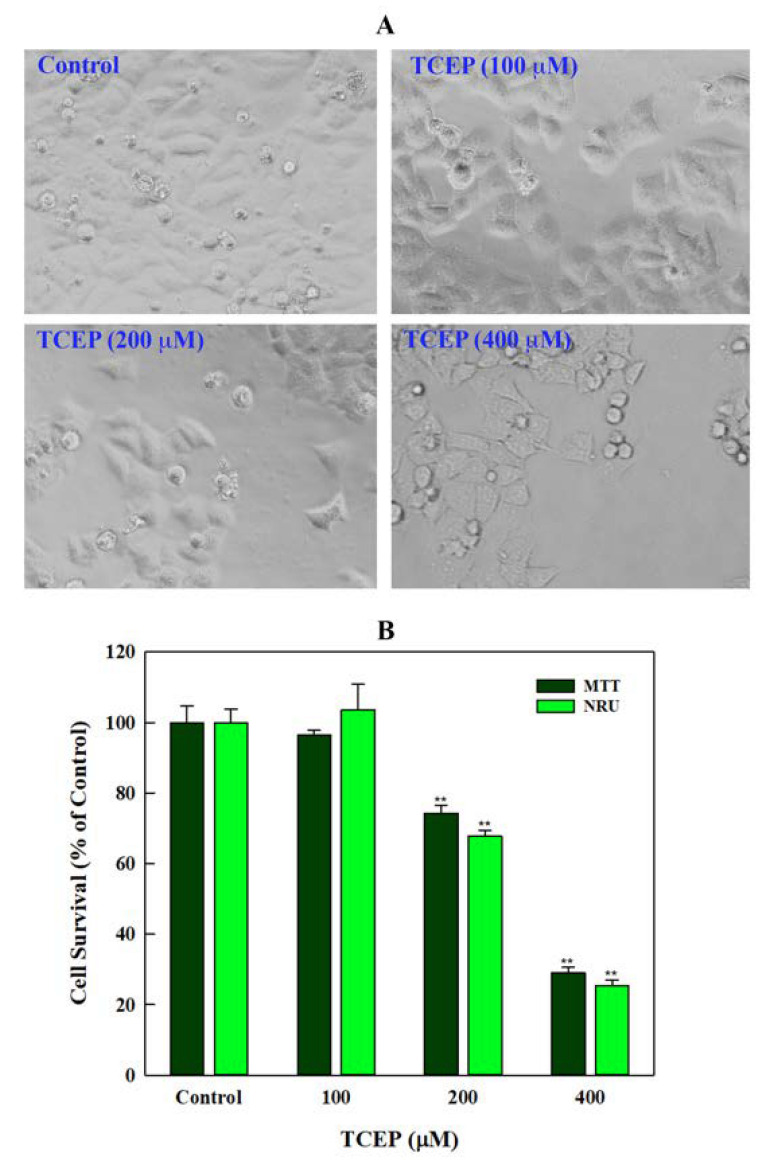
Effect of TCEP on cell survival after prolonged exposure (3 days): (**A**) HepG2 cells exhibited morphological changes, adjacent cell gaps, and detachment after TCEP exposure. (**B**) Quantitative analysis of cytotoxicity using MTT and NRU assays. Each histogram in panel B is the mean ± S.D. of three experiments done in triplicate wells. ** *p* < 0.01 versus control.

HepG2 cells exposed to TCEP for 3 days exhibited proliferation inhibition, which manifested as development of gaps among the neighbouring cells and their detachment from the culture plates (Figure 1A). Cytotoxic responses in HepG2 cells were quantitated by the mitochondrial dehydrogenase enzyme based MTT assay. Presence of TCEP (200, 400 μM) in cell culture media for 3 days significantly decreased the survival of HepG2 to 25.68% and 70.92% (Figure 1B), while the lowest concentration of TCEP (100 μM) showed non-significant reduction (3.44%) in HepG2 survival. Subsequently, TCEP-exposed cells were assessed for lysosomal toxicity using the NRU assay. Similar to MTT assay responses, the NRU assay also showed a significant reduction in HepG2 survival to 32.23% and 75.57% after exposure to TCEP at higher concentrations (200 and 400 μM). The lowest concentration (100 μM) showed a non-significant (3.53%) reduction in cell survival (Figure 1B).

### 3.2. Quantitation of DNA Damage

**Figure 2:**
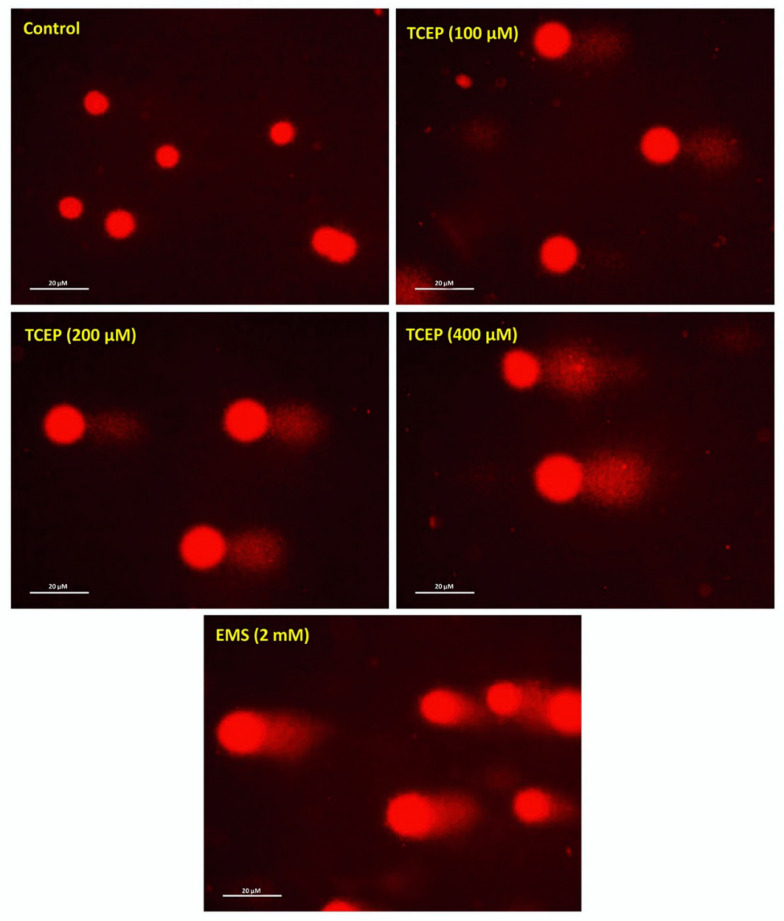
Comet assay exhibiting DNA strand breaks in TCEP (3 days) treated HepG2 cells: epifluorescence images showing broken DNA in the form of tails electro-stretched from the nuclei. Undamaged cells showing round nuclei. EMS: ethyl methanesulfonate used as a positive control. Fluorescence microscope was used to capture images at a magnification of 20×.

Comet assay data showed extensive DNA damage in the HepG2 cells upon TCEP exposure. In relation with the Olive tail moment (OTM) value of 0.43 in controls, HepG2 cells grown in the presence of 100, 200, and 400 μM of TCEP (3 days) revealed 7.1-, 11.7-, and 20-fold greater OTM values. Among the other parameters of comet assay (i.e., tail length TL), 1.9-, 2.3-, and 2.8-fold increases in TL were found in cells grown in the presence of 100, 200, and 400 μM of TCEP, while control cells showed 43.84 µm of TL. The percent tail intensities (TI) in TCEP (100, 200, 400 μM) treated cells were 3.3-, 4.8-, and 8.1-fold. Relatively, control cells showed only 2.3 (%) TI (Table 1). The representative comet images captured after TCEP exposure validate DNA breaks (Figure 2).

### 3.3. Flow Cytometric Data

#### 3.3.1. HepG2 Cell Cycle Dysfunction by TCEP

**Figure 3:**
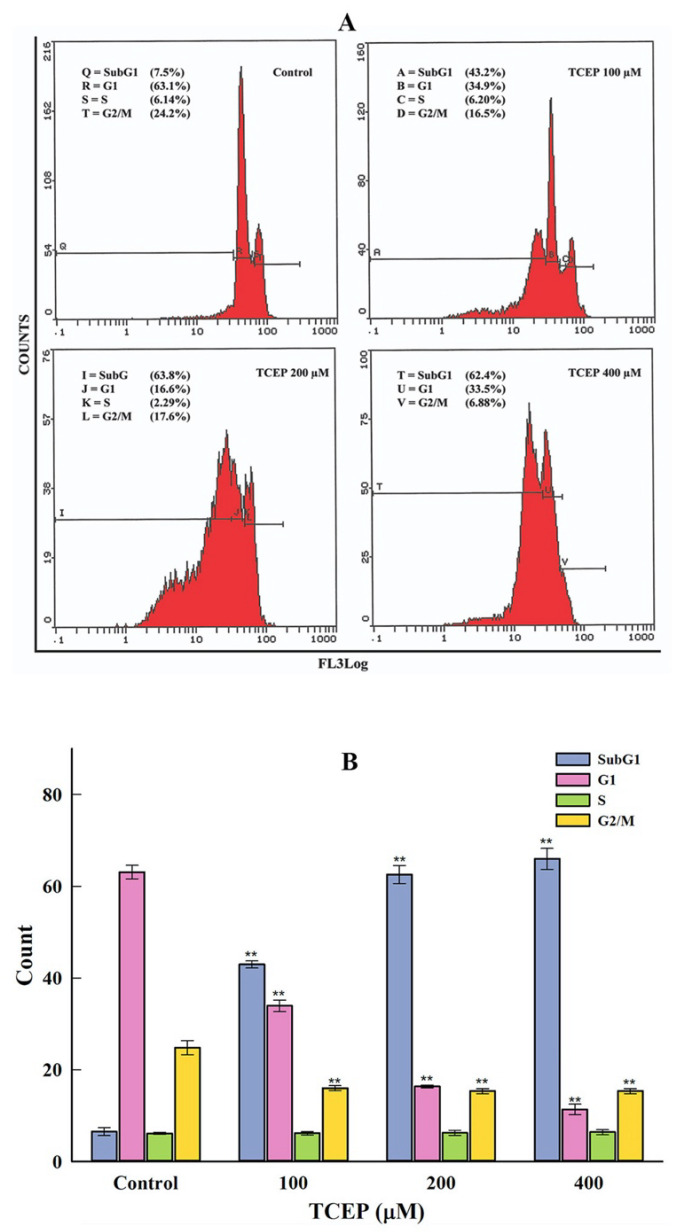
HepG2 cell cycle alterations after TCEP (3 days) treatment analyzed by a flow cytometer. (**A**) Flow cytometric images showing concentration-dependent upsurge of apoptotic peak (subG1). Inset texts showing % of cell population present in different phases (subG1, G1, S, G2/M) of the cell cycle analyzed by drawing markers. (**B**) Histograms are the cumulative values (mean ± S.D.) of cell populations appearing in the subG1, G1, S, and G2/M phases obtained from three independent experiments done in duplicate wells. ** p < 0.01 versus control.

HepG2 cells exposed to TCEP for 3 days showed significant disturbances in the cell cycle phases. The typical flow cytometric images of cell cycle showed an increment in the apoptotic subG1 peak in TCEP-exposed HepG2 cells (Figure 3A). Relative to average data of the background apoptotic peak in the control (6.56 ± 0.87%), HepG2 cells grown in the presence of 100, 200, and 400 μM of TCEP (3 days) displayed a considerable upsurge in the subG1 peak, quantitated as 42.96 ± 0.77%, 62.58 ± 1.07%, and 65.96 ± 2.34%, respectively (Figure 3B).

#### 3.3.2. TCEP Induced Oxidative Stress and Esterase Activity

**Figure 4:**
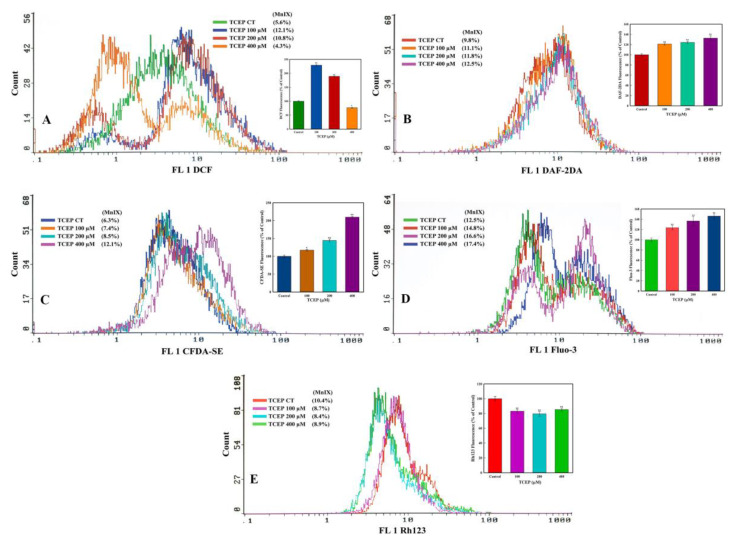
Flow cytometric analysis of ROS, NO, esterase, Ca^2+^ influx, and mitochondrial dysfunction in HepG2 cells after 3 days of exposure with TCEP: (**A**–**D**) Representative flow cytometric images showing fluorescence enhancement of dyes DCF, DAF-2DA, CFDA-SE, and Fluo-3 indicating higher ROS, NO, esterase, and Ca^2+^ influx in HepG2 cells. (**E**) Decline in Rh123 fluorescence indicates perturbance of mitochondrial membrane potential (*ΔΨm*). Insets in each panel are the cumulative values (mean ± S.D.) obtained from three independent experiments done in duplicate wells. * *p* < 0.05, ** p < 0.01 versus control. MnIX: mean intensity of dye on the *x*-axis.

TCEP exposure for 3 days induced much higher ROS production in the HepG2 cells. The flow cytometric overlay graph showed a logarithmic shift in DCF fluorescence in TCEP-treated cells, measured as mean intensity (MnIX), and specified intracellular ROS production (Figure 4A). Relative to 100% DCF fluorescence in the untreated control, 229.1% and 189.2% higher fluorescence of DCF was found in cells exposed to 100 and 200 μM of TCEP, while 400 μM exposed HepG2 cells showed 23.2% decline in DCF fluorescence (Figure 4A inset). TCEP-exposed cells also showed a substantial amount of NO production, which is obvious with the shift of MnIX values towards greater log scale (Figure 4B). HepG2 cells grown in the presence of 100, 200, and 400 μM of TCEP (3 days) showed 121.1%, 124.2%, and 132.7% increment over 100% fluorescence of DAF2 in control (Figure 4B inset). An increased intracellular esterase activity was recorded in the HepG2 cells upon TCEP exposure, as evident from the logarithmic shift of CFDA fluorescence peaks on the log scale (Figure 4C). Compared to the 100% fluorescence of CFDA in control cells, TCEP (100, 200, 400 μM) exposed HepG2 cells demonstrated 117.3%, 144.3%, and 209.8% fluorescence enhancement (Figure 4C inset).

#### 3.3.3. Ca^2+^ Influx and *ΔΨm* Alteration by TCEP

TCEP triggered a large upsurge in Ca^2+^ influx in the HepG2 cells, which was recorded as a prominent shift in Fluo-3 fluorescence peaks towards greater values on the logarithmic scale of the flow cytometric overlay image (Figure 4D). Relative to 100% fluorescence of Fluo-3 in the control, TCEP (100, 200, 400 μM) exposed cells showed fluorescence augmentation of 121.1%, 124.2%, and 132.7%, respectively (Figure 4D inset). On the other hand, effects of TCEP on HepG2 *ΔΨm* were manifested as decreased fluorescence (MnIX values) of Rh123 dye on the flow cytometric overlay graph (Figure 4E). Compared to Rh123 fluorescence (100%) in the control, HepG2 exposure to TCEP (100, 200, and 400 μM) decreased dye fluorescence by 83.0%, 79.4%, and 85.3% (Figure 4E inset).

### 3.4. Transcriptomic Changes in HepG2 by TCEP Exposure

**Figure 5:**
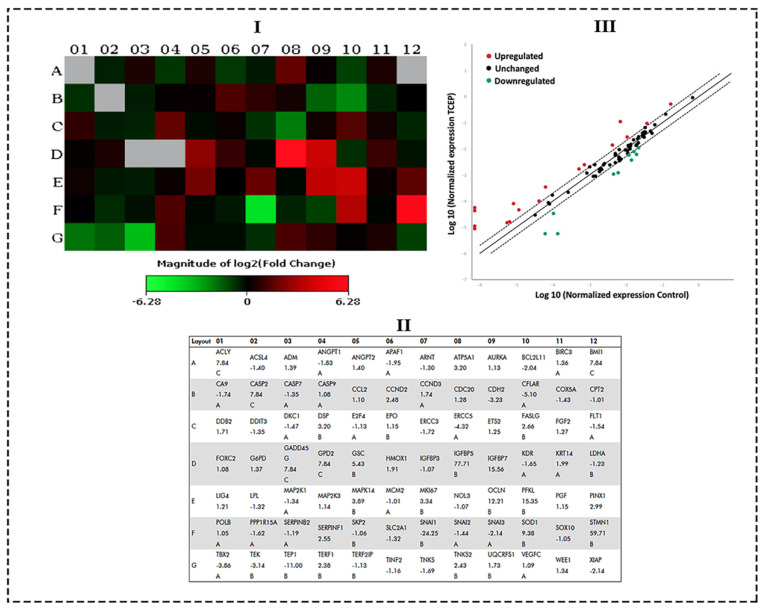
qPCR array of human cancer pathway genes in HepG2 cells treated with TCEP (100 μM, 3 days): (**I**) Heat map depicting the activation of upregulated (red boxes) and downregulated (green boxes) genes in the array plate. Gray boxes are undetermined genes. (**II**) Layout of qPCR array plate with the corresponding genes. (**III**) Scatterplot exhibiting genes which were upregulated and downregulated at a threshold value of >2.0-fold regulation (---). Upregulated genes (red dots), downregulated genes (green dots), unchanged (black dots).

qPCR array data obtained from TCEP (100 μM, 3 days) exposed HepG2 cells showed a number of genes belonging to the human cancer pathways were activated. Figure 5I,III show the heat map and scatter plot data, indicating the upregulated and downregulated genes in HepG2 cells. Fold change values for all genes after TCEP exposure are depicted in Figure 5II. Among the prominently upregulated genes were Insulin-like growth factor binding protein 5 (*IGFBP5*, 77.71-fold), Stathmin 1 (*STMN1*, 59.71-fold), Insulin-like growth factor binding protein 7 (*IGFBP7*, 15.56-fold), Phosphofructokinase, liver (*PFKL*, 15.35-fold), Occludin (*OCLN*, 12.21-fold), Superoxide dismutase 1, soluble (*SOD1*, 9.38-fold), Goosecoid homeobox (*GSC*, 5.43-fold), Mitogen-activated protein kinase 14 (*MAPK14*, 3.89-fold), Antigen identified by monoclonal antibody Ki-67 (*MKI67*, 3.34-fold), Desmoplakin (*DSP*, 3.20-fold), ATP synthase, H^+^ transporting, mitochondrial F1 complex, alpha subunit 1, cardiac muscle (*ATP5A1*, 3.20-fold), PIN2/TERF1 interacting, telomerase inhibitor 1 (*PINX1*, 2.99-fold), Fas ligand (TNF superfamily, member 6) (*FASLG*, 2.66-fold), Serpin peptidase inhibitor, clade F (alpha-2 antiplasmin, pigment epithelium derived factor), member 1 (*SERPINF1*, 2.55-fold), Cyclin D2 (*CCND2*, 2.48-fold), Tankyrase, TRF1-interacting ankyrin-related ADP-ribose polymerase 2 (*TNKS2*, 2.43-fold), and Telomeric repeat binding factor (NIMA-interacting) 1 (*TERF1*, 2.38-fold) (Figure 5I,III). Genes that were downregulated in the cancer pathway after TCEP exposure (3 days) were Snail homolog 1 (Drosophila) (*SNAI1*, -24.25-fold), Telomerase-associated protein 1 (*TEP1*, -11.00-fold), CASP8 and FADD-like apoptosis regulator (*CFLAR*, -5.10-fold), Excision repair cross-complementing rodent repair deficiency, complementation group 5 (*ERCC5*, -4.32-fold), T-box 2 (*TBX2*, -3.86-fold), Cadherin 2, type 1, N-cadherin (neuronal) (*CDH2,* -3.23-fold), TEK tyrosine kinase, endothelial (*TEK*, -3.14-fold), X-linked inhibitor of apoptosis (*XIAP*, -2.14-fold), Snail homolog 3 (Drosophila) (*SNAI3*, -2.14-fold), and BCL2-like 11 (apoptosis facilitator) (*BCL2L11*, -2.04-fold) (Figure 5I,III).

## 4. Discussion

The suitability of OPFRs to delay fire incidences has led to their progressive utilization as an additive chemical in several consumer products such as plastics, paints, foams, electronics, and construction and building materials. During the lifespan of a product, OPFR’s additive properties makes them liable to leach into the surrounding environment due to improper usage, disposal, as well as production. Such effects precede OPFRs into environmental settings and the human body via inhouse or outside environmental exposure, as also evidenced by the occurrence of OPFRs in the breast milk of lactating mothers, human urine, and potable water samples [8,10,29]. Widespread existence of OPFRs in the environment raises alarming concerns on its environmental toxicity and human health hazards. In this regard, bioaccumulated TCEP was found in human fat and detected in body fluids, signifying grave health concerns [7,11]. Some in vitro and in vivo test models have affirmed liver toxicity due to TCEP exposure, and they categorized TCEP as a carcinogenic chemical [13,14,17].

There is still a paucity of evidence on how TCEPs induce transcriptomic alterations related to cancer pathways in hepatic cells. Such information will help to recognize the comparable consequences in humans exposed to TCEP and provide governing bodies with information to define TCEP exposure limits. Hence, in the current study HepG2 cells were selected due to their genetic similarities to the normal hepatocytes. HepG2 cells are regarded as a good in vitro model for hepatotoxic assessment of chemicals [24]. Cell viability or proliferation measurements are considered as good markers of cell health [30]. Consequently, along this line we quantitated cell survival, which has confirmed that HepG2 cells exposed to TCEP for shorter time periods (1 and 2 days) did not exhibit reduced proliferation (data not shown). We found that TCEP (3 days) exposure at the highest concentrations (200 and 400 μM) exhibited significant reductions in HepG2 cell survival, indicating cytotoxic effects. TCEP-exposed HepG2 cells exhibited higher cytotoxic effects in the NRU assay as well, specifying greater lysosomal damage and resulted in reduced dye uptake, feasibly done via impairment of lysosomal acid phosphatase activity [31]. A comparable trend of cytotoxic effects in human peripheral blood mononuclear (PBM) and human lung (A549) cells was also recorded upon their treatment for 1 day with TCEP (1000 μM) and 2 days with TCP (300 μM) [21,22]. Chang liver cells also showed cellular anomalies upon exposure to TCEP, exhibiting cell cycle arrest, impairment of mitochondrial functionality, downregulation of SIRT3 proteins, and upsurge in Ca^2+^ level [32]. These repercussions strongly validate our data, indicating that the toxicological threshold of TCEP is efficacious at high concentrations and after prolonged exposure. Concerning human exposure to TCEP, inhalation has been attributed as a primary route. Nonetheless, inadvertent ingestion of dust containing TCEP may provide an alternative path to enter the body, where its bioaccumulation may elicit analogous cytotoxic effects, as also observed in the MTT and NRU experiments.

Interactions of toxic chemicals with biological macromolecules, metabolic reactions, receptor binding, and cell membranes have been shown to affect cell health [30]. Hence, to verify the role of TCEP-induced cytotoxicity and cell health, we further evaluated its effect on DNA integrity. By means of the comet assay, the present study explicates that TCEP in a concentration-dependent manner introduced strand breaks in DNA. Broken DNA in the matrix of microgel was found similar to that reported with higher doses (1–10 mg/kg) of OPFR-exposed earthworm coelomocytes and HepG2 cells exposed to TCP (400 μM) [28,33]. The quantum of DNA damage specifies towards the impairment of DNA repair machinery to rectify the damage. Failure of the repair system instigates apoptotic death, distinctly evidenced by an increased percentile of HepG2 cells in the subG1 apoptotic phase. The observed trend is in agreement with the responses of A549 cells, which exhibited caspase-dependent apoptosis after 48 h of TCEP treatment [22]. ROS and NO both have the ability to generate peroxynitrite, and both can induce DNA strand breaks and trigger apoptosis [34,35].

In this line, flame retardants (6-OHBDE-47) as well as OPFRs (TCEP, TCP) have demonstrated their selective toxicity by ROS generation [22,28,31]. The current study also showed TCEP-exposed HepG2 cells have nitric oxide (NO) and ROS buildups. The esterase level was also augmented in the exposed cells. Similar to our findings, greater levels of ROS and NO were quantitated in rat neurons, A549, and HepG2 cells as a consequence of BDE-209, TCEP, and TCP exposure [22]. ROS and NO have overwhelming roles in causing mitochondrial membrane damage, thereby eliciting Ca^2+^ influx to trigger apoptosis [34,36]. In addition, *ΔΨm* fluorescence decreased upon TCEP treatment, implying an impairment of mitochondrial membrane structure and thereby instigating alterations of ATP production to elicit apoptotic cell death [37,38]. We further quantitated the expression of cancer pathway genes in HepG2 cells exposed to noncytotoxic concentrations of TCEP. A proper rationale to choose low concentrations for transcriptome analysis primarily encompasses the noncytotoxic effects of TCEP observed in MTT and NRU assays; secondly, it also unravels the putative role of TCEP in activating cancer pathway genes at low concentrations. On the other hand, higher concentrations of TCEP in our study specify a wider scenario of toxicological repercussions in human liver cells upon accidental or workplace exposure. HepG2 cells exposed to TCEP exhibited activation of genes in the cellular senescence pathway. Especially, *IGFBP5*, *SOD1*, and *IGFBP7* genes were highly upregulated, perhaps due to the heavy DNA damage and apoptosis after TCEP exposure. Upregulation of *IGFBP5* has also been recorded during DNA damage and apoptosis in mammary epithelial cells [39,40]. Higher expression of *SOD1* reflects the consequence of greater ROS and NO by TCEP, which was beyond the mitigating capacity of cells to protect them from oxidative stress and cell death [28]. *IGFBP5* and *IGFBP7* are among the 16 IGFBP family members in which *IGFBP*5 binds strongly to IGFs, while *IGFBP7* binds with low affinity [41]. Within this cohort, *IGFBP*5 modulates metabolism, differentiation, migration, invasion, and cell growth [42,43,44]. On the other hand, *IGFBP7* synchronizes cellular senescence, proliferation, differentiation, cell adhesion, and angiogenesis in varying types of cells. Additionally, *IGFBP7* functions as a tumor suppressor gene in different cancer types [41,45,46,47,48]. The upregulation of *IGFBP5* and *IGFBP7* certifies that these genes gravely affected the proliferation and also participated in the senescent phenomena in TCEP-exposed HepG2 cells, finally triggering apoptosis [49].

*STMN1* in the cell cycle pathway, and *MAPK14, PFKL, ATP5A1* in the cell metabolism pathway, were highly upregulated after TCEP exposure. Being a hallmark in cancer prediction, the *STMN1* gene plays key roles in cell cycle regulation, proliferation, and progression of cancer. Accordingly, disturbances to the cell cycle with subsequent appearance of subG1 apoptotic peaks in TCEP-exposed cells legibly justifies the upregulation of *STMN1*. On top of that, *STMN1* is phosphorylated by MAPKs during intracellular signaling [50]. In this relation, upregulation of *MAP2K14* in HepG2 cells signifies its divergent role to regulate *STMN1* expression. *PFKL* is a key gene in glycolysis; its high expression serves as a switch for glycolysis in rapidly proliferating cancer cells. *PFKL* activation often promotes proliferation and metastasis through activation of the Warburg effect in cancer cells [51]. Upregulation of *PFKL* implies an aberrant metabolism in TCEP-exposed HepG2 cells, perhaps due to greater glucose demand, which needs to be thoroughly investigated in future studies. Moreover, *ATP5A1* is directly connected with the mitochondrial ATP synthase and the catalysis of ATP synthesis. Its upregulation in HepG2 cells upon TCEP exposure unequivocally stipulates the *ΔΨm* dissipation data, and it certifies the fact that TCEP affected the operation of oxidative phosphorylation and may augment to mitochondrial encephalopathy [52].

*OCLN* was upregulated in the epithelial-to-mesenchymal transition (EMT) pathway. On the other hand, *SNAI1* and *CDH2* were found downregulated after TCEP exposure. *OCLN* has been related with execution of the extrinsic apoptosis pathway through the stimulation of death-inducing signaling complexes of death receptors [53,54]. In line with our observations, *OCLN* was upregulated and involved in the apoptotic sensitivity of keratinocytes [55]. *SNAI1* is member of the *Snail* gene family, encoding the zinc finger proteins, and primarily functions as a transcriptional repressor and protects cells from apoptosis [56,57,58]. *SNAI1* is a master regulator of EMT, mainly suppressing the expression of E-cadherin (*CDH1*), which is a cell adhesion molecule that mediates Ca^2+^-dependent cell–cell homophilic interactions via adherens junctions [59]. Cellular detachment and gaps between HepG2 cells upon TCEP exposure correspond well with the downregulation of N-cadherin (*CDH2*), indicating its functional suppression to preserve intracellular adhesion between cells [60]. Downregulation of mesenchymal marker N-cadherin (*CDH2*) also stipulates the putative involvement of *SNAI1* and other EMT transcriptional factors to regulate its expression either alone or together.

## 5. Conclusions

MTT data showed decreased survival in HepG2 cells after TCEP exposure at higher concentrations. These effects substantiate the likelihood that TCEP interferes with the active metabolism of viable cells, leading to an increase in the percentage of dead cells. HepG2 cells exposed to TCEP also showed decreased survival in the NRU assay, further validating the fact that TCEP affected the architecture of lysosomal membranes to influence the accumulation NR dye in lysosomes. Moreover, comet assay data showed that TCEP-exposed HepG2 cells exhibited an increase in DNA damage visualized as a comet tail. The DNA damage data unequivocally corroborate the genotoxic capability of TCEP in HepG2 cells. Cell cycle analysis of TCEP-treated HepG2 cells demonstrated an increase in the apoptotic peak (subG1), confirming the onset of apoptotic cell death. Flow cytometric analysis of TCEP-exposed HepG2 cells exhibited an increase in ROS, NO, Ca^2+^ influx, and esterase level. In addition, TCEP-exposed HepG2 cells displayed a reduction in *ΔΨm*. Collating the flow cytometric data, it can be surmised that an increase in apoptotic cells is an outcome of mitochondrial impairment and severe oxidative stress in HepG2 cells as a consequence of TCEP exposure. HepG2 transcriptomic variations undoubtedly conclude that TCEP harbors the possibility to activate key genes in human cancer pathways. Nonetheless, our in vitro study warrants further validation in in vivo test models to analyze the hepatotoxic effects of TCEP.

## Figures and Tables

**Table 1:** DNA damage in HepG2 cells after 3 days of TCEP exposure, analyzed using different parameters of alkaline comet assay.

Groups	Olive Tail Moment (OTM) (Arbitrary Unit)	Tail Length (TL)(µm)	Tail Intensity (TI) (%)
Control	0.43 ± 0.03	43.84 ± 1.83	2.35 ± 0.13
EMS (2 mM)	11.49 ± 0.72 **	107.43 ± 2.61 **	33.81 ± 1.65 **
TCEP (µM)			
100	3.09 ± 0.24 **	84.39 ± 3.70 **	7.91 ± 0.32 **
200	5.05 ± 0.59 **	105.19 ± 3.86 **	11.28 ± 0.81 **
400	8.64 ± 0.77 **	123.71 ± 2.51 **	19.04 ± 0.40 **

Data are the mean ± S.D. of three independent experiments done in duplicate wells. ** *p* < 0.01 versus control; EMS: ethyl methanesulfonate used as a positive control.
